# 16th International congress on antiphospholipid antibodies task force report on antiphospholipid syndrome laboratory diagnostics and trends

**DOI:** 10.1177/09612033231211820

**Published:** 2023-11-07

**Authors:** Tatsuya Atsumi, Cecilia B Chighizola, Yuichiro Fujieda, Ian Mackie, Massimo Radin, Robert Roubey, Maria Laura Bertolaccini

**Affiliations:** 1 Department of Rheumatology, Endocrinology and Nephrology, Faculty of Medicine, Hokkaido University, Sapporo, Japan; 2 Unit of Pediatric Rheumatology, ASST G. Pini - CTO, Department of Clinical Sciences and Community Health, University of Milan, Milan, Italy.; 3 Department of Haematology, Haemostasis Research Unit, University College London, London, UK; 4 Department of Clinical and Biological Sciences, and SCDU Nephrology and Dialysis, S. Giovanni Bosco Hospital, Center of Research of Immunopathology and Rare Diseases - Coordinating Center of Piemonte and Valle d’Aosta Network for Rare Diseases, Turin, Italy; 5 Division of Rheumatology, Allergy & Immunology, Thurston Arthritis Research Center, University of North Carolina, Chapel Hill, NC, USA; 6 Academic Department of Vascular Surgery, School of Cardiovascular and Metabolic Medicine & Sciences, 4616 King’s College London, London, UK

**Keywords:** Phosphatidylserine-dependent antiprothrombin antibodies, antibodies to domains of β_2-_glycoprotein-I, anti-β_2_Glycoprotein-I antibodies

## Abstract

Classification criteria for antiphospholipid syndrome (APS) require IgG or IgM isotypes of the anticardiolipin (aCL) antibodies, anti-β2 glycoprotein I (anti-β2GPI) antibodies, and/or the lupus anticoagulant (LA) to satisfy the laboratory disease definition. Over the past 20 years, non-criteria antiphospholipid antibodies (aPL) directed to other proteins of the coagulation cascade (i.e. prothrombin and/or phosphatidylserine–prothrombin complex) or to some domains of β2GPI have been proposed. This task force concentrated and reviewed the literature on data including aPS/PT, antibodies to domain 4/5 of β2GPI and the newly described antibodies to protein/HLA-DR complex. In addition, we discussed testing of LA in the ‘new’ oral anticoagulants’ era and the value of triple positivity in the risk assessment of aPL. The conclusions were presented at a special session during the 16^th^ International Congress on aPL, Manchester, UK, September 2019.

## Introduction

Classification criteria for antiphospholipid syndrome (APS) require IgG or IgM isotypes of the anticardiolipin (aCL) antibodies, anti-β2-glycoprotein I (anti-β2GPI) antibodies, and/or the lupus anticoagulant (LA) to satisfy the laboratory criterion for disease definition.^1^ Over the past years several ‘non-criteria’ antiphospholipid antibodies (aPL), directed to proteins of the coagulation cascade (i.e., prothrombin and/or phosphatidylserine–prothrombin complex) or to specific domains of β2GPI have been focused upon.^2,3^

The task force on antiphospholipid syndrome laboratory diagnostics and trends focused and reviewed the literature on data including aPS/PT, antibodies to domain 4/5 of β2GPI and the newly described antibodies to protein/HLA-DR complex. In addition, we discussed testing of LA in the ‘new’ oral anticoagulants’ era and the value of triple positivity in the risk assessment of aPL. The conclusions were presented at a special session during the 16^th^ International Congress on aPL, Manchester, UK, September 2019 and updated previous to this publication.

## Phosphatidylserine-dependent Antiprothrombin Antibodies (aPS/PT)

Many reports show the clinical utility of phosphatidylserine-dependent antiprothrombin antibodies (aPS/PT) assay in the diagnosis of APS. The inclusion of aPS/PT antibodies as a laboratory criterion of APS has been previously considered by our task force and deemed unwarranted because of poor standardization of the available assays and because reproducibility of the strong correlations between aPS/PT and APS manifestations needed confirmation in larger studies.^4^

Anti-phosphatidylserine/prothrombin (aPS/PT) antibodies are a low affinity heterogeneous class of antibodies directed against a complex of negatively charged phospholipid, other than cardiolipin and prothrombin (PT). The methodology for their detection has improved over the years and currently aPS/PT antibodies are identified by enzyme immunosorbent assays using prothrombin in complex with phosphatidylserine in the presence of calcium. Calcium ion aids the binding of prothrombin to phosphatidylserine inducing major conformational changes to the prothrombin structure which, in turn, exposes cryptic or neo-epitopes that act as target for aPS/PT.^5^ The site of binding on the prothrombin molecule is still under research and it may be possible that different types of antibodies can recognize different sites of the prothrombin molecule.^6^ So far, it has been established that aPS/PT and aPT represent two different types of antibodies that can be present concurrently in some cases.^7^ Prothrombin 1 and fragment 1 and fragment 1+2 have been reported as potential antigens recognized by antiprothrombin antibodies, suggesting that the dominant epitopes are likely to be located near the phospholipid-binding site of the prothrombin molecule.^5^ A recent study by Chinnaraj and colleagues used prothrombin mutants and identified 2 subpopulations of aPS/PT, namely type I and type II, which engage fragment 1 of prothrombin at different epitopes.^8^

Testing for aPS/PT antibodies has been proposed as an additional tool to be considered when investigating a patient suspected of having APS, particularly in the absence of routine aPL positivity,^2,9^ or as a part of risk assessment strategies.^10^ aPS/PT represent in fact a stronger risk factor for thrombosis, both arterial and/or venous, than aPT^11^ and in combination with LA and anti-β2GPI offer the best diagnostic indication of APS.^12^ This is supported by a recent study by Pengo and colleagues, that demonstrated that the search for aPS/PT antibodies along anti-β2GPI antibodies, in patients positive for LA, might be useful to identify two distinct subgroups of patients at different risk of thromboembolic events.^13^

In addition, patients with triple positivity for LA, anti-β2GPI and aPS/PT have been shown to be at a higher risk of developing thromboembolic events, risk even higher than that seen for the ‘classical’ aCL, anti-β2GPI, and LA triple positivity.^12,14^

An early systematic review evaluated papers from 1988 to 2013 and assessed the correlation between aPT and aPS/PT antibodies and the risk of thrombosis.^11^ Among 10 studies on aPS/PT, comprehensive of 1775 patients and 628 controls, eight of them confirmed the association with thrombosis, but only seven compared the ORs. When performed, multivariate analysis sustained aPS/PT correlation with thrombosis and venous events. The correlation was confirmed in a more recent systematic review^15^ that analyzed the studies on aPS/PT and their correlation with clinical manifestations of APS from 2012 to 2019. Briefly, the patient population included 1219 patients classified as APS according to Sidney criteria,^1^ 285 patients with isolated persistently positive aPL and 1397 patients with a clinical suspicion of APS.

Twelve studies, including 1888 patients, analyzed the association between aPS/PT antibodies and thrombosis, observing a statistically significant association between aPS/PT IgG/IgM positivity and thrombotic events (mean OR 6.8 [95% CI 3.18–16.4], *p* < 0.05), confirmed when analyzing aPS/PT IgG (mean OR 6.7 [95% CI 3.04–21.6], *p* < 0.05) and aPS/PT IgM (mean OR 4.35 [95% CI 1.54–17.77], *p* < 0.05) separately. Seven studies, including 1388 patients, evaluated the association between aPS/PT antibodies and pregnancy morbidity. When pooled together, a statistically significant association between any pregnancy morbidity and aPS/PT IgG/IgM positivity (mean OR 10.6 [95% CI 3.54–35.38], *p* < 0.05), particularly aPS/PT IgG positivity (mean OR 6.7 [95%CI 3.04–21.6], *p* < 0.05) was found.

Overall, the current available data highlights the strong association between aPS/PT and the clinical manifestations of APS. With the available level of evidence, aPS/PT testing can be considered as a robust test applicable in the investigation of patients suspected of APS, also beyond the research settings.

## Antibodies against domains of β2GPI: a focus on domain 4/5

β2 glycoprotein I (β2GPI) provides the main antigen targeted by aPL, the diagnostic serum biomarkers and pathogenic effectors of APS. β2GPI is believed to exert a relevant biological function, as suggested by the evolutionary conserved structure of this molecule. Although β2GPI-deficient mice are apparently healthy, recent findings suggest that β2GPI may serve as a bridge between the innate immune system and the coagulation cascade.^16^ Indeed, β2GPI can engage lipopolysaccharide (LPS), being potentially deputed to remove endotoxin from the circulation.^17^ In addition, β2GPI can affect the activation of complement cascade: on one hand, it enhances the degradation of C3 by factor I, on the other it activates the lectin pathway by binding directly to the mannose-binding lectin. The latter interaction is mediated by the glycan content of β2GPI, which is approximately 20%.^18,19^ In turn, the complement lectin pathway can upregulate coagulation factors thus promoting the activation of coagulation.^20^ Furthermore, β2GPI directly modulates coagulation by exerting both a procoagulant and an anticoagulant action. The latter includes the prevention of platelet aggregation induced by ADP and von Willebrand factor, the inhibition of thrombin, factor Xa and tissue activator of plasminogen. However, procoagulant mechanisms prevail: β2GPI inhibit procoagulant protein C, displace anticoagulant Annexin A5 and prevent the formation of thrombomodulin/thrombin complexes.^16^ Even the opposite interaction exists. Plasmin can inactivate β2GPI by cleaving the molecule in a motif shared with plasminogen (named kringle domain); interestingly, such inactivation can be further augmented by heparin.^16^

β2GPI is composed of five domains (D): D1 to D4 share a homologous structure which consists in 60 amino acids also found in complement control proteins, while D5 is aberrant. It contains an allosteric disulphide bond, which easily undergoes redox modifications, and a loop of lysine residues, which – due to its positively charge – is deputed to interaction with anionic phospholipids (PL), including cardiolipin (CL), as well as with coagulation and complement factors.^16^ More than 90% of circulating β2GPI adopts a circular conformation, with D1 interacting with D5. β2GPI opens to a J-shaped conformation upon binding to CL and LPS or following changes in pH and oxidative state. This is extremely relevant, as the opening of the molecule leads to the exposure of cryptic epitopes.^21^

From early days in APS research, efforts have focused on the characterization of the reactivity of anti-β2GPI antibodies from APS patients against the different portions of the β2GPI molecule. The first pioneer studies envisaged linear peptides, the more recent ones employ conformational epitopes and more sophisticated techniques. As detailed in Table 1, available studies documented a polyreactivity of anti-β2GPI antibodies from APS patients against different domains of the molecule. However, it is now widely acknowledged that a cryptic and conformation-dependent structure in the N-terminal D1 of the molecule provides the most relevant epitope involved in β2GPI/anti-β2GPI antibody binding.^22–31^ Consistently, antibodies against D1 have been extensively documented to exert a pathogenic role in mediating both thrombotic and obstetric complications of the syndrome.^28,32–34^ Anti-D1 antibodies are the prevalent autoantibody subsets among APS patients, being detected across the different cohorts in 40%–88% of subjects with thrombotic APS and 17%–84% of women with obstetric complications.^35^ Such wide range in the positivity rates of anti-D1 antibodies might be ascribed to the composition of study populations and to the detection methods.

**Table 1.** Reactivity against the different domains of β2 glycoprotein I of antiphospholipid antibodies from patients with antiphospholipid syndrome.

Author, year (REF)	D1	D2	D3	D4	D5
Hunt, 1994^84^					X
Wang, 1995^85^				X	
Igarashi, 1996^22^	X	X	X	X	
Yang, 1998^86^					X
George, 1998^87^				X	
Iverson, 1998^23^	X				
Blank, 1999^88^			X	X	
Reddel, 2000^24^	X				
McNeeley, 2001^25^	X				
Iverson, 2002^26^	X				
Shoenfeld, 2003^27^	X	X	X	X	X
Kasahara, 2005^89^				X	
De Laat, 2005^28^	X				
Ioannou, 2007^29^	X				
De Laat, 2011^30^	X				
De Moerloose, 2017^31^	X				
Serrano, 2019^90^			X	X	X

D: domain.

Besides D1, other studies have focused on the characterization of the reactivity of antibodies against D4 and D5 of β2GPI.^36–39^ The available studies used a research ELISA kit from INOVA Diagnostics (San Diego, CA, USA), which employs recombinant D4 and D5 bound to the polystyrene microwell plate under conditions preserving the native state. These studies are concordant in describing a low prevalence of anti-D4/5 antibodies (below 30%) in patients with APS (Table 2). Furthermore, the reports reject any association between anti-D4/5 antibodies and clinical manifestations of APS, including vascular events and obstetric manifestations (Table 2). Notably, among patients with aPL positivity associated with other systemic autoimmune rheumatic disease (SARD), the positivity rate of anti-D4/5 antibodies is similar (ranging between 16.7% and 35.3%).^36,37^ Likewise, in these studies, anti-D4/5 antibody reactivity is not associated with thrombotic complications.^36^ Interestingly, subjects with high-risk aPL profile (namely, two or three positive aPL tests) present lower anti-D4/5 titres and a lower anti-D4/5 positivity rate when compared to those with single aPL positivity.^38,39^ Consistently, patients with anti-D4/5 antibodies tend to display a single positivity for anti-β2GPI antibodies with negative aCL and LA.^39–43^ Anti-D4/5 antibodies do not react in the β2GPI-dependent aCL assay, with a magnitude effect of binding inhibition dependent on the β2GPI concentration, the hypothesis being that anti-D4/5 antibodies do not react against β2GPI complexed with CL due to the spatial proximity of the two binding sites.^41–43^

**Table 2.** Antibodies against domains 4 and 5 of β2 glycoprotein I and clinical manifestations of antiphospholipid syndrome.

Author, year ^REF^	Isotype	*N*	Study population	Thrombosis		Pregnancy morbidity		Controls	
				Positivity rate	Association	Positivity rate	Association	*N*	Positivity rate
**Despierres, 2014** ^36^	IgA	59	SLE	35.3%	No association	N.R.	N.R.	N.R.	N.R.
**Andreoli, 2015** ^37^	IgG	87	anti-β2GPI + PAPS	19.6%	No association	16.1%	No association	30	40%
**Pengo, 2015** ^38^	IgG	65	anti-β2GPI + subjects	N.R.	No association	N.R.	N.R.	40	N.R.
**Chighizola, 2018** ^39^	IgG	108	anti-β2GPI + PAPS	29.1%	No association	29.5%	No association	27	37%

N: number; N.R.: not reported; SLE: systemic lupus erythematosus; APS: antiphospholipid syndrome; PAPS: primary anti-phospholipid syndrome.

The above discussed data clearly suggests that anti-D4/5 antibodies could be useful in assessing risk in individuals with anti-β2GPI antibodies with data suggesting that their presence imply a lower risk. The other side of the coin relates to the so-called asymptomatic aPL carriers or subjects with clinical manifestations not related to aPL positivity. Indeed, in these populations, anti-β2GPI antibodies preferentially recognize D4 and D5, as documented in many studies. As detailed in Table 2, aPL carriers (in other words, individuals who are persistently positive for aPL but do not develop any manifestation of the syndrome) present a higher positivity rates and higher titres of anti-D4/5 compared to APS patients.^20–22^ Antibodies purified from subjects with isolated positivity for anti-D4/5 antibodies have been recently shown to react against D5 by means of recombinant domains in the immunoenzymatic assay.^43^ Other investigators characterized the domain reactivity of anti-β2GPI antibodies from subjects with non-APS condition, localizing the epitope alternately in D4 or D5. Using domain-deleted mutants, Iverson and colleagues observed that anti-β2GPI IgA from 29 patients with atherosclerotic syndrome preferentially target D4.^21^ In another study, anti-β2GPI antibodies of IgG and/or IgM isotypes were detected in 39% of 176 lepromatous patients. The authors performed in vitro experiments using β2GPI deleted mutants and 8C3, a monoclonal antibody that binds to D1, and concluded that anti-β2GPI antibodies in subjects with leprosy react against D5.^42^ Paediatric subjects are an additional population of interest, since anti-β2GPI antibodies are frequently detected in healthy children. It is possible that the *de novo* production of these autoantibodies follows the ubiquitous environmental diffusion of β2GPI. In a cohort of 93 children with different allergic diseases, a high frequency of anti-β2GPI IgG was found in those with atopic dermatitis (42%). The observation that proteolytic cleavage of PL-binding site in the C-terminal loop in D5 abolished antibody binding to β2GPI suggested that the antigenic site resides in close vicinity.^41^ A subsequent study found an even higher positivity rate of anti-β2GPI in 33 children with atopic dermatitis (54.5%), with a polarization towards D4/5 reactivity (33%). Anti-D4/5 antibodies have been detected in 21 of 57 one-year-old healthy children born to mothers with SARD (36.8%), whereas anti-D1 antibodies tested positive in nine children (15.7%).^44^

The observation that anti-D4/5 antibodies do not react with β2GPI complexed with CL might imply that such autoantibody subpopulation does not react against cell-bound β2GPI and thus are not able to elicit a pathogenic potential.^27^ As a matter of fact, when the pathogenic potential of antibodies purified from subjects with isolated positivity for anti-D4/5 antibodies in mediating vascular occlusion was explored in LPS-treated Wistar rats, these antibodies failed to promote thrombosis. This was evaluated as vessel occlusion in mesenteric vessels using intravital microscopy and was significantly different from what was observed with anti-D1 IgG.^43^ In vitro experiments showed that anti-D5 antibodies bind significantly less to D5 after incubation with β2GPI at increasing concentrations, suggesting its interaction with β2GPI in soluble form. These results led to formulate the hypothesis that anti-D5 might antagonize the procoagulant activity of anti-D1 antibodies by competing for β2GPI binding. As approximately 15% of APS patients display positivity for both anti-D1 and anti-D5 IgG,^37^ these observations might exert important clinical implications. Indeed, due to the interaction of anti-D5 IgG with soluble β2GPI, it is tempting to speculate that anti-D5 IgG might prevent β2GPI binding to target cells, thus antagonizing the procoagulant activity of anti-D1 antibodies. The magnitude of this competitive effect might depend on the relative anti-D1 and anti-D5 antibody levels. This intriguing hypothesis has been partially explored in patients by calculating the ratio between anti-D1 and anti-D4/5 antibodies (the so-called ‘anti-D1:anti-D4/5 ratio’). In two studies, the relevance of this tool has been evaluated in anti-β2GPI IgG positive patients subgrouped upon clinical presentation: thrombotic and/or obstetric APS, SARD, and asymptomatic aPL positivity. Interestingly, employing two research ELISAs, it was evinced that an anti-D1:anti-D4/5 ratio above 1.5 is predictive of systemic autoimmunity (APS and SARD).^37^ Other authors detected anti-D1 and anti-D4/5 antibodies by a novel line immunoassay; an anti-D1:anti-D4/5 ratio above 4.6 could significantly distinguish APS patients from subjects with SARD.^45^ In another study including exclusively patients with primary APS, anti-D1:anti-D4/5 ratio above 2.1 yielded an odds ratio of 2.7 for APS diagnosis, with a sensitivity of 62% and a specificity of 63%.^39^

Overall, current evidence rejects any association between D4/5 epitope specificity and both thrombotic and obstetric manifestations of APS. Consistently, available data negate a role for anti-D4/5 antibodies in the pathological processes of APS. However, the hypothesis of a protective role of anti-D4/5 antibodies warrants further confirmation. Available evidence comes from a single group, and some data are controversial. When rats were treated with three different autoantibody preparations (anti-D1 antibodies, anti-D4/5 antibodies, IgG from normal healthy subjects) no difference in vascular deposition of β2GPI could be evidenced at immunofluorescence of mesenteric tissue, a finding that conflicts with the hypothesis that anti-D4/5, by binding to β2GPI, might prevent its endothelial localization. At present, anti-D4/5 antibodies might be regarded as a second-line test to be reserved to anti-β2GPI antibody positive subjects to refine the process of stratification of the hazard of future clinical events. Future works should aim at clarifying the clinical significance of anti-β2GPI antibodies not reacting against D1 and D4/5, which is the case in approximately one fourth of APS subjects.

## Antibodies against protein/HLA-DR complex

Anti-β2GPI/Human Leukocyte Antigen (HLA)-DR antibodies has been reported as a new aPL test based on a novel mechanism of antigen presentation.^46^ The specific APS susceptible HLA-DR alleles influence the cell-surface expression of β2GPI/HLA-DR complexes. β2GPI/HLA-DR complexes are considered as major target antigens for autoantibodies in patients with APS.

Genome-wide analysis has confirmed that major histocompatibility complex (MHC) class II loci showed strong association with susceptibility to many autoimmune diseases.^47^ MHC class II molecules are primarily expressed by professional antigen presentation cells, such as dendritic cells, macrophages, B cells. MHC class IIα- and β-chains assemble in the endoplasmic reticulum (ER) and form a complex with the invariant chain.^48^ The invariant chain-MHC class II heterotrimer is transported through the Golgi to the MHC class II compartment, either directly and/or via the plasma membrane. Consequently, peptides derived from the endocytic compartment are presented as antigen to CD4+ T cells. In general, misfolded proteins localized in the ER are degraded promptly inside cells. The novel theory, however, is that misfolded proteins are rescued from protein degradation in the ER2 and transported to the cell surface by MHC class II molecules as ‘neo-self-antigens’. Jiang et al.^46^ found that misfolded proteins can associate with MHC class II molecules in the ER instead of the invariant chain, depending on allelic polymorphisms in MHC class II genes. In addition, ‘neo-self-antigens’ activate the antigen-specific B cells,^49^ suggesting that these complexes of misfolded proteins with MHC class II molecules are considered major targets of autoantibodies in multiple autoimmune diseases.^50^

aPL bind to some part of β2GPI/HLA-DR complexes which are transported to the cell surface without degradation. aPL may recognise an epitope on the β2GPI/HLA-DR complexes as well as phospholipid binding β2GPI. Anti-β2GPI/HLA-DR antibodies are detected by a ‘cell-based assay’ using 293T cells co-transfected with β2GPI and HLA-DR.^51^ Since almost half of the APS patients were single positive for anti-β2GPI/HLA-DR antibodies, while negative for anti-β2GPI and aCL, β2GPI/HLA-DR complexes may expose unique epitopes that are not present on plate-bound β2GPI or β2GPI/CL complexes. Interestingly, the expression levels of β2GPI on cell surface were different between HLA-DR7 and HLA-DR8. The comprehensive analysis of HLA-DR alleles revealed that, in addition to HLA-DR7, HLA-DR4 was transporting high levels of β2GPI to the cell surface and, conversely, several other HLA-DR alleles were transporting very little β2GPI. HLA-DR7 or HLA-DR4 has been reported as APS susceptibility alleles,^52^ suggesting that specific HLA-DR alleles might be associated with expressing ‘neo-self-antigens’ as well as susceptibility allele to APS. Unlike T cell receptors, HLA-DR allele itself does not affect the autoantibody binding to β2GPI/HLA-DR complexes. Sera from 120 patients with APS were examined and over 80% of APS patients showed anti-β2GPI/HLA-DR7 antibodies.^51^ Considering these results, using β2GPI/HLA-DR7 complex as an assay antigen is enough to analyze the prevalence of anti-β2GPI/HLA-DR antibodies in patients with APS.

Anti-β2GPI/HLA-DR antibodies are also associated with obstetric complication in patients with APS. In fact, β2GPI/HLA-DR complexes were found in uterine decidual tissues from patients with APS.^51^ Recently, by conducting prospective, multicentre, cross-sectional study, Tanimura et al.^53^ reported that women with unexplained recurrent pregnancy loss (RPL) had anti-β2GPI/HLA-DR antibodies. RPL was defined as the loss of ≧ 2 pregnancies and β2GPI/HLA-DR7 complex was used as an antigen to detect anti-β2GPI/HLA-DR antibodies in these women. Of the 227 women with RPL, 45 (19.8%) tested positive for aPL such as LA, aCL, or anti-β2GPI and 52 (22.9%) tested positive for anti-β2GPI/HLA-DR antibodies. Of these, 35 (67.3%) were single-positive. Among the women with unexplained RPL, 24 (19.8%) were positive for anti-β2GPI/HLA-DR antibodies. Of the 112 women who did not meet criteria for APS, 21 (18.8%) were positive for anti-β2GPI/HLA-DR antibodies. These results suggest that anti-β2GPI/HLA-DR antibodies may be useful in classifying women with unexplained RPL into obstetric APS.

Anti-β2GPI/HLA-DR antibodies have a potential as disease-specific antibodies in APS. However, the presence of β2GPI/HLA-DR complexes in the human body remains to be investigated. β2GPI is produced not only by hepatocytes, but also by endothelial cells and placental villous tissue.^54,55^ Non-immune cells strongly express MHC class II molecules in response to stimulation from interferon γ (IFNγ), but do not express costimulatory molecules required for the induction of T cell responses, such as CD80 or CD86. Stimulated non-immune cells can activate B cell directly. β2GPI/HLA-DR complexes can be expressed on the cell surface during infection or inflammation and could stimulate B cells to produce autoantibodies. This aberrant HLA expression in APS would explain the pathogenicity of autoimmune disease, leading to novel therapeutic approaches.

## Lupus anticoagulant and new anticoagulants

The ISTH survey on LA testing^56^ showed that 70.3% of participants thought that LA testing should not be performed in patients receiving direct oral anticoagulants (DOAC). Among the participants, 17% suggested that testing could be performed in the trough period, 11% after pre-treatment of the sample with commercial DOAC adsorbent or antidote preparations, while 2.7% felt that LA testing may be undertaken in some circumstances in patients on DOACs during the peak concentration period. This indicates that there is considerable uncertainty about what action to take in patients receiving DOACs.

There are numerous publications demonstrating that DOACs interfere with LA testing, causing false positive or false negative results^57–66^ and there are particular problems with direct thrombin inhibitors. There are a variety of potential ways around the problems:

1. Wait until the patient has stopped DOAC or switch to a different anticoagulant
2. Collect sample in the trough period
3. Use a LA test that is not affected by DOAC
4. Neutralise/remove DOAC

Stopping the anticoagulant or waiting until the patient has finished their course of anticoagulant treatment are not usually practical. LA detection could influence the drug choice, intensity of anticoagulation and duration of treatment. Stopping anticoagulation or switching to a different anticoagulant (bridging therapy) could put the patient at risk of adverse events. Low molecular weight heparin is less likely to affect LA tests (most commercial reagents contain heparin neutralisers effective up to about 1 IU/mL). There is also a potential for patient confusion about any altered dosing or change of medication type if the anticoagulant is changed to facilitate blood tests.

Testing for LA during the trough period would appear to be a suitable approach, since anticoagulant levels would be too low to have much influence on the tests. However, false positive dilute Russel’s viper venom time (DRVVT) results have been reported in trough even at very low (<50ng/mL) rivaroxaban levels.^61,65^ It is also difficult to be certain that the patient is in the trough period, as there may have been errors or variability in the time of taking medication. One answer might be to assay the DOAC, but the sensitivity varies between assays and the minimum concentration of each DOAC type that affects LA tests is not known (and different LA tests vary in their sensitivity to DOACs). External quality assurance studies have shown a small degree of inter-laboratory variability in DOAC assays (10%–12%), but the greatest differences between reagents were observed for rivaroxaban, especially at concentrations above 100ng/mL, where the cv was 10%–15% and there was a difference of about 20ng/mL between methods.^67^ Many laboratories have set up their assays to measure samples at peak DOAC levels and the lower detection limit of their method may be only 50ng/mL. A recent publication^68^ studied 60 venous thromboembolism patients receiving DOAC (30 rivaroxaban, 30 apixaban) during the trough period and found mean levels of 23ng/mL (range <18–68) and 42ng/mL (19–99) respectively.93% of rivaroxaban and 40% apixaban patients had a false positive DRVVT due to a marked effect of the DOAC on the screening part of the test. Similarly, 40 and 30% of patients had false positive silica clotting time and 17 and 20% showed false positive APTT ratio comparing LA sensitive and insensitive reagents. In addition, LA results did not necessarily correlate with the drug level, even when patients appeared to be truly in the trough period.

Dabigatran appears to affect most LA tests, since it is a direct thrombin inhibitor and all well recognised LA diagnostic tests employ a coagulation end point method. For direct factor Xa inhibitors, the Taipan/Ecarin venom clotting time ratio appears to be suitable,^58,59,69,70^ but the reagents are currently only available from one supplier (Diagnostic Reagents Ltd, Thame, UK) and the method has not been validated for apixaban. The method requires further standardisation and wider assessment before it can be adopted into routine clinical practice (ISTH guidelines).

Drugs have been developed that are therapeutic antidotes and neutralise DOACS. Adexanet alpha has been used in vitro but does not fully neutralise rivaroxaban and therefore does not normalise the clotting time,^71^ but in vitro addition of idarucizumab (a therapeutic humanized antibody fragment) neutralized dabigatran.^72^ The regular use of these therapeutic agents in vitro in clinical diagnostic laboratories is likely to be difficult and cost prohibitive. It would be difficult to know whether neutralization/removal had occurred and the relevant DOAC would probably have to be assayed after sample treatment. It is also unknown whether residual neutralizing agent can interfere in the DOAC assay.

A more successful approach appears to be with the use of activated charcoal products to remove DOAC, although this is not without problems. There are two commercial branded products, DOAC-STOP (Haematex Research) and DOAC-Remove (5-Diagnostics) that have been widely studied.^72–76^ DOAC-STOP was shown to reduce DOAC levels to <30ng/mL in 60%–100% of VTE patient samples and known APS patient samples remained LA positive after treatment. However, some studies have reported 5%–15% false LA positives, which may be partly due to incomplete DOAC removal, particularly if drug levels are >350ng/mL. DOAC-Remove is generally effective, but one study showed that DOAC was incompletely removed in 4% of samples. An unbranded activated carbon product (Norit Carbomix, Kela Pharma) has also been shown to be effective, but a few false positive LA results were observed. The authors suggested that the normal plasma used for mixing studies and cut-off determination should also be treated.^77^

In summary, the various activated charcoal products are not completely problem free. Some of the studies have used DOAC spiked samples rather than performing ex vivo studies and even then, the number of patient samples has been limited. DOAC plasma levels were not always assayed after sample treatment to ensure adequate removal. Further studies are underway with larger amounts of removal agent which may resolve some of the problems. Unless the DOAC removal methods are sanctioned by LA test reagent/analyser manufacturers, the technique may be deemed as manipulation and create regulatory and accreditation problems, meaning that full, local validation exercises will have to be undertaken before adoption into routine clinical laboratory practice.

## Assessing ‘Triple Positivity’

Antiphospholipid antibody (aPL) ‘triple positivity’ is defined as positivity in the three most used clinical aPL laboratory tests: (1) aCL IgG or IgM, medium or high level, (2) anti-β2GPI IgG or IgM, medium or high level, (3) LA performed in accordance with international standards. It is well-established and broadly accepted that aPL ‘triple positivity’ is a high-risk phenotype for the clinical manifestations of the antiphospholipid syndrome (APS).

**Figure 1.**
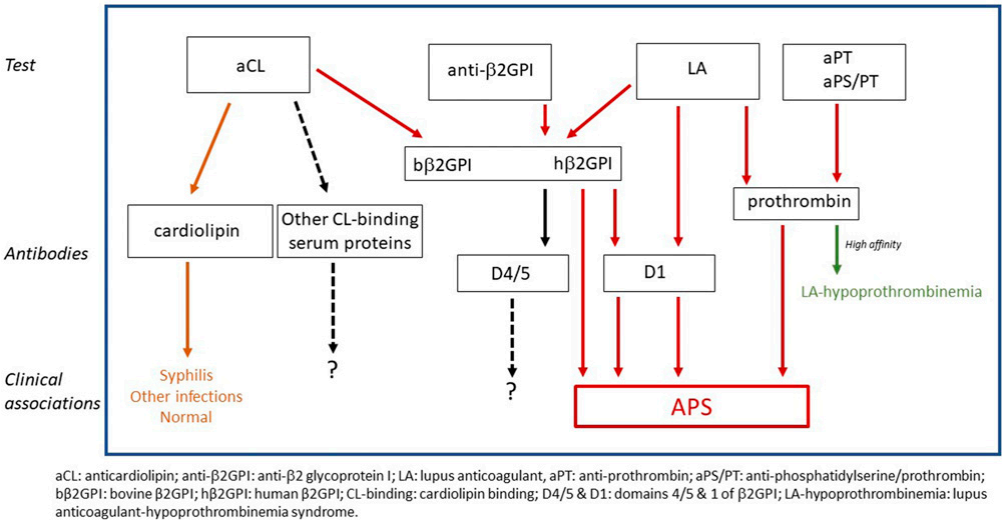
Relationship of aPL clinical tests, antibodies detected, and associated clinical manifestations.

To understand the nature of ‘triple positivity’ it is important to recognize that it is defined based on clinical tests and not on antibody specificity. Historically this has been a source of confusion. Figure 1 schematically shows the relationship among major clinical laboratory tests, the antibodies detected in each test, and the clinical associations of these antibodies. Typical aCL assays can detect antibodies of various specificities. Although purified cardiolipin is the intended antigen, bovine serum is typically used as the blocking agent/sample diluent and cardiolipin-binding proteins in bovine serum (notably, β2GPI) can also serve as target antigens. Thus, aCL assays can detect antibodies binding directly to cardiolipin (seen in patients with syphilis, certain other infections, and occasionally in normal individuals), antibodies to bovine β2GPI (associated with APS), and possibly antibodies to other cardiolipin-binding proteins in bovine serum (without known clinical associations). Anti-β2GPI assays detect antibodies to purified human β2GPI (associated with APS). Research has shown that antibodies responsible for lupus anticoagulant activity (in the setting of APS) can be directed against β2GPI or prothrombin. Available data also suggest that antibodies to D1 of β2GPI have LA activity^28^ while the value of antibodies to D4/5 is still being investigated. In contrast to the relatively lower affinity anti-prothrombin antibodies in APS patients, very high affinity anti-prothrombin antibodies, which are quite rare, can be associated with hypoprothrombinemia and a bleeding disorder. Anti-prothombin and aPS/PT immunoassays which detect anti-prothrombin antibodies are emerging.

aCL and anti-β2GPI tests detect largely overlapping subsets of antibodies to β2GPI. In APS patients the concordance of these two tests is high. By definition, ‘triple positive’ patients are concordant. There are several reasons why there may be discordant results in these two tests. Firstly, antibodies recognizing cardiolipin in aCL assays are not detected in anti-β2GPI tests. Secondly, although anti-β2GPI in most APS patients are reactive with both human and bovine β2GPI there are rare patients with antibodies specific for human, but not bovine, β2GPI. Thirdly, some discordance may be due to the epitope specificity of anti-β2GPI antibodies and the differential display of epitopes in the two assays.

LA tests are coagulation-based assays, not immunoassays. These tests detect certain antibodies based on the antibodies’ interference with a clotting reaction. A considerable body of research demonstrates that anti-β2GPI or anti-prothrombin antibodies exert LA activity if these antibodies can form high avidity cross-linked phospholipid-bound antigen/antibody complexes in in vitro coagulation assays thereby inhibiting these coagulation reactions by decreasing the available anionic phospholipid surface. There are two major factors that appear to determine whether anti-β2GPI and/or anti-prothrombin antibodies have LA activity. The first is epitope specificity. For example, it is likely that antibodies to D1 of β2GPI can cross-link phospholipid-bound β2GPI since D1 is exposed when the protein is bound to a membrane. In contrast, antibodies to D4/5 of β2GPI may not be able to cross-link bound β2GPI because these domains are not exposed or less exposed on the membrane-bound molecule. The second key factor, and one that is sometimes overlooked, is antibody concentration. Simply put, in contrast to the exquisite analytical sensitivity of ELISAs and other immunoassays, antibody inhibition of an in vitro coagulation reaction requires high antibody concentration. This has been clearly demonstrated with both monoclonal and polyclonal antibodies.^78–81^ For monoclonal antibodies, LA activity requires 100- to 1000-fold higher concentrations compared to detection in ELISAs. Using polyclonal IgG purified from 2 APS patients with high levels of IgG anti-β2GPI, we observed that LA activity required 5 to 10 times the IgG concentration that was strongly positive in an anti-β2GPI ELISA. These observations suggest that LA activity and, therefore, ‘triple positivity’ are proxies for high antibody titer. Additionally, positive LA tests can reflect the combined effect of all relevant antibodies in a given specimen (aCL, anti-β2GPI, anti-prothrombin, all isotypes of each specificity) and do not indicate a single antibody profile. A positive LA can be due to anti-β2GPI in some patients, anti-prothrombin antibodies in other patients, and perhaps to both specificities in still others. In a cohort of 254 patients from the Antiphospholipid Syndrome Collaborative Registry, LA activity was associated with higher levels of IgG aCL and IgM aCL, and this association was more pronounced when IgG and IgM levels were combined.^81^

More recently the association of antibody levels, LA activity, and ‘triple positivity’ was evaluated in a group of patients enrolled in the APS ACTION Registry Database. Complete core lab data (aCL, anti-β2GPI, and interpretable LA results) were available from 325 patients. Most of these patients (281, 86%) were LA positive; 80 patients (25%) were ‘triple positive’. An ‘aPL load’ was crudely calculated as the sum of four test results (IgG aCL, IgM aCL, IgG anti-β2GPI, IgM anti-β2GPI). Note that this calculation does not include aPS/PT, anti-PT, IgA aCL, or IgA anti-β2GPI. As shown in Table 3, ‘aPL Load’ was strongly associated with LA positivity and ‘triple positivity’.

**Table 3.** ‘aPL Load’, LA positivity and ‘triple positivity’.

A	LA	*N*	Average ‘aPL Load’
	Negative	44	63.1 ± 78.4
	Positive	281	134.1 ± 123.0

B	‘aPL Load’ (quintiles)	*N*	LA+	% LA+
	Q1 (0–25)	64	46	72%
	Q2 (26–50)	66	52	79%
	Q3 (51–117)	65	59	91%
	Q4 (118–220)	66	64	97%
	Q5 (221–665)	64	60	94%
	All subjects	325	281	86%

C	‘Triple positivity’	*N*	Average ‘aPL Load’
	Not ‘triple positive’	245	72.2 ± 64.3
	‘Triple positive’	80	284.6 ± 110.3

*p* < 0.0001, Mann-Whitney test.

X^2^ for trend = 21.23, *p* < 0.0001.

*p* < 0.0001, Mann-Whitney test.

It has long been recognized that the risks for thrombosis, pregnancy morbidity and mortality, and other clinical manifestations of APS are associated with higher aPL titers. Higher risk is also associated with LA positivity and with ‘triple positivity’. It is likely that LA activity requires both epitope specificities that allow for the cross-linking of membrane-bound antigens, for example, antibodies to D1 of β2GPI, as well as high levels of such antibodies. Importantly, the factors that contribute to LA activity in vitro appear to play an important role in the pathogencity of aPL in vivo. For example, dimerized D5 of β2GPI (which mimics membrane-bound β2GPI cross-linked by antibodies to D1) has been shown to upregulate monocyte procoagulant activity mimicking the effects of anti-β2GPI.^82^

Scoring strategies that combine and weight aPL tests, such as the antiphospholipid score^83^ and the Global APS Score^10^ (the latter of which also considers other risk factors) may prove useful in applying the concept of aPL ‘load’ to clinical evaluation.
